# Evaluation of repositories for sharing individual-participant data from clinical studies

**DOI:** 10.1186/s13063-019-3253-3

**Published:** 2019-03-15

**Authors:** Rita Banzi, Steve Canham, Wolfgang Kuchinke, Karmela Krleza-Jeric, Jacques Demotes-Mainard, Christian Ohmann

**Affiliations:** 1 Istituto di Ricerche Farmacologiche Mario Negri IRCCS, Milan, Italy; 2 Canham Information Systems, Surrey, UK; 3 Coordination Centre for Clinical Trials, Heinrich Heine University, Düsseldorf, Germany; 4 Karmela Krleza-Jeric – IMProving Access to Clinical Trial data (IMPACT) Observatory, Mediterranean Institute for Life Sciences (MedILS), Split, Croatia; 5 European Clinical Research Infrastructure Network (ECRIN), Paris, France; 6 European Clinical Research Infrastructure Network (ECRIN), Düsseldorf, Germany

**Keywords:** Clinical studies, Data sharing, Data repositories, Individual-participant data, Metadata

## Abstract

**Background:**

Data repositories have the potential to play an important role in the effective and safe sharing of individual-participant data (IPD) from clinical studies. We analysed the current landscape of data repositories to create a detailed description of available repositories and assess their suitability for hosting data from clinical studies, from the perspective of the clinical researcher.

**Methods:**

We assessed repositories that enable storage, sharing, discoverability, re-use of the IPD and associated documents from clinical studies using a pre-defined set of 34 items and publicly available information from April to June 2018. For this purpose, we developed an indicator set to capture the maturity of the repositories’ procedures and their suitability for the hosting of IPD. The indicators cover guidelines for data upload and data de-identification, data quality controls, contracts for upload and storage, flexibility of access, application of identifiers, availability of metadata, and long-term preservation.

**Results:**

We analysed 25 repositories, from an initial set of 55 identified as possibly relevant. Half of the included repositories were generic, i.e. not limited to a specific disease or clinical area and 13 were launched in the last 8 years. The sample was extremely heterogeneous and included repositories developed by research funders, infrastructures, universities, and editors. All but three repositories do not apply a fee for uploading, storage or access to data. None of the repositories completely demonstrated all the items included in the indicator set, but three repositories (Dryad, Drum, EASY) met – fully or partially – all items. Flexibility of data-access modalities appears to be limited, being lacking in half of the repositories.

**Conclusions:**

Our evaluation, though often hampered by the lack of sufficient information, can help researchers to find a suitable repository for their datasets. Some repositories are more mature because of their support for clinical dataset preparation, contractual agreements, metadata and identifiers, different modalities of access, and long-term preservation of data. Further work is now required to achieve a more robust and accurate system for evaluation, which in turn may encourage the sharing of clinical study data.

**Trial registration:**

Study protocol available at https://zenodo.org/record/1438261#.W64kW9Egrcs.

**Electronic supplementary material:**

The online version of this article (10.1186/s13063-019-3253-3) contains supplementary material, which is available to authorized users.

## Background

Data sharing is increasingly being recognized as a key requirement of scientific research. Within clinical research, data sharing can enhance reproducibility and the generation of new knowledge, but it also has an ethical dimension. This is because sharing data from clinical studies better respects the generosity of study participants by increasing the utility of the data they provide and, thus, the value of their contribution. Calls for making clinical study data sharing the norm have been promoted by several stakeholders [1–5] and, though perhaps more slowly than in other research fields, participant-level clinical studies data are now becoming increasingly available for further research use [6].

Data repositories have the potential to play an important role in the effective and safe sharing of clinical study data because they can provide a stable, long-term home for the data, improve the security and quality of archiving through active data curation, increase the discoverability of data through the application of metadata schemes, and facilitate the processes of request and transfer of data from generators to users, as well as tracking data utilisation [7]. The central role of data repositories in improving data sharing in clinical research was recognised in the context of a consensus exercise that developed principles and recommendation on sharing and reusing individual-participant data (IPD) from clinical trials [5]. As of October 2018, the DataCite database, re3data, lists more than 2000 data repositories, hosting data and documents from different scientific disciplines, although only a tiny minority of them appear to host clinical study data [8].

Several funders require that a data management plan that includes provision for data sharing is submitted along with the grant application and some have established dedicated data repositories [9–11]. Increasingly, medical editors require that data underlying the results presented in journal articles are made available to other researchers and suggest repositories where data can be stored and identified [12–15]. Some universities and research institutions have established data-sharing policies and related data archives, mainly to improve dissemination and outreach activities. These institutional initiatives support any type of research data and clinical study data may, therefore, also be potentially stored and accessed through these systems. In 2014, Clinicalstudydatarequest.com (CSDR) [16] and Yoda [17] were launched. These platforms are not data repositories as such – instead they facilitate the interaction between data generators (usually industry sponsors) and data users, allowing access to specific datasets made available by the study sponsors within a controlled analysis environment.

Despite the advent of these repositories, initiatives, portal systems, and the gradual increase in the sharing of data from clinical studies, the role of data repositories appears, so far, to be relatively limited [18, 19]. For instance, IPD meta-analyses are usually conducted by contacting the clinical investigators who ran and analysed the original studies, who usually retain the data themselves. It may be that many existing repositories are not adequate for clinical studies, as, for instance, they do not fully support the forms of restricted access often required for IPD datasets. On the other hand, clinical researchers and sponsors, especially in academia, may not be familiar with the resources available for long-term data management, and thus the storage and sharing options available to them.

The information about the different repositories able to store clinical research ‘data objects’ – the generic term used to refer to any document or dataset in digital form – may be useful in the development of the data-sharing plan. This is a document or a section in the protocol reporting where, when and how to store and make available for sharing the IPD and associated documents (e.g. study protocol, case report form, data management plan).

The purpose of this study was to provide detailed information to sponsors and researchers about the data repositories available to them. Considering mainly those repositories that would be accessible to non-commercial researchers, for the deposition of IPD and related documents from clinical studies, we describe the options for managing data, associated costs, and available data-access mechanisms. We provide narrative descriptions of repository features, focussing on data submission and storage, description, curation, preservation, and processes for accessing data, so far as that information is publicly available. We then estimate the suitability of each repository for clinical study data.

## Methods

The protocol of this evaluation is available on Zenodo (https://zenodo.org/record/1438261#.W64kW9Egrcs).

### Identification and selection of repositories

We identified repositories, publicly accessible via the Internet, combining formal and informal iterative searching. The work was based on the activities of previous working groups within CORBEL and the IMProving Access to Clinical Trial data (IMPACT) Observatory [5, 20–22]. We broadly searched the web using keywords such as ‘open data and clinical trials’, ‘clinical trial data sharing’, ‘repository’, ‘database’, ‘clinical research’ and ‘study results’ as well as the re3data database [8]. Additional information was retrieved from the relevant literature, personal contacts, meetings, and conferences. This approach was not intended to be a systematic search, but it was considered the most appropriate and feasible in evaluating a field as dynamic and heterogenous as data sharing. We included repositories with details available in the English language, so that we may have missed some repositories, e.g. some operating exclusively in Asia.

We focussed on repositories developed by public or private not-for profit institutions (e.g. universities, research institutions) that enable storage, sharing, discoverability, and re-use of IPD from clinical studies and related study documents. We excluded clinical trial registries/databanks and patient registries, as they usually host health data originating in a non-research context, i.e. clinical practice, and trial registries as they contain protocol information and summary results (aggregate data). We excluded repositories developed by pharma companies as the focus was on repositories allowing the upload of data from non-commercial studies. We also excluded metadata portals, (like CSDR and Yoda) as they do not store data themselves. We initially focussed on repositories able to handle a broad spectrum of clinical data, but the importance of disease-specific repositories, such as those promoted by National Institute of Health (NIH)-funded institutions in the United States, soon became clear, and so we included representatives of such repositories within the sample.

### List of items assessed

For each repository included in the analysis, we collected and assessed a pre-defined set of 34 items (Additional file 1). These features were proposed and agreed among the group of authors and built on the previous work and discussion within the CORBEL and the IMPACT Observatory projects. They were meant to provide a general characterisation of the repository, and included aspects used to help assess the repository’s relative maturity and its suitability for clinical research data. We split the items into four groups: (1) General parameters, including scope, funding and establishment date, and description of the repository; (2) Data upload and storage – in particular the practical steps required for depositing IPD and related documents, including potential costs; (3) Data management and access – both quantitative and qualitative descriptions of the types of access provided and of the data, including an indication of the approximate number of clinical studies stored in the repository, as well as the type of material available; and ( 4) Discoverability – the details of metadata schemas applied and how those metadata are made available.

### Data collection

From April to June 2018, pairs of authors independently assessed eligibility for each candidate repository, collecting data and filling in a pre-defined form that spanned the 34 data items. The data extracted were then checked for consistency and discrepancies, which were resolved by discussion among the four authors involved in data extraction (RB, SC, CO, WK). The sources of information were the public pages of the repository websites, including usage contracts, usage guidelines, Q&A pages, FAQs, support pages, privacy policy pages, etc., and those documents available after simple registration, as well as reports, publications, and other publicly available materials about the repositories, as press releases, and presentations. We did not contact the repositories directly to gather missing information or clarifications.

### Data analysis

The information collected was summarised and used to estimate the suitability of the repository for hosting clinical study data. Certification systems for data repositories exist, such as the CoreTrustSeal certification launched by the merging of World Data Systems and Data Seal of Approval [23–25]. However, they provide basic requirements for repositories and we considered them to be not sufficiently specific for health research data. For the purpose of this study we selected the following eight features from the 34 data points and used them as indicators of suitability: (1) guidelines for data upload and storage, (2) support for data de-identification, (3) data quality controls, (4) contracts for upload and storage, (5) availability of metadata, (6) application of identifiers, (7) flexibility of access, and (8) repository long-term preservation. The choice of these indicators was based on considering what we thought would be the most important features for clinical researchers looking to deposit data in a repository. Some of them signal the general level of maturity of a repository’s systems, for instance, data quality controls and the availability of metadata. Others, especially guidelines for data de-identification and flexibility of access, are particularly important for sensitive data such as IPD. For each of the included repositories, two authors independently judged each item in the ‘suitability indicator’ set based on the information collected in the previous phase. Each item was classified as ‘demonstrated’, ‘partially demonstrated, ‘not demonstrated, or ‘missing or partial information available’. Additional file 2 provides further details on the criteria used for each indicator and the detailed criteria used to differentiate each category. Discrepancies were discussed and solved by consensus.

## Results

**Fig. 1:**
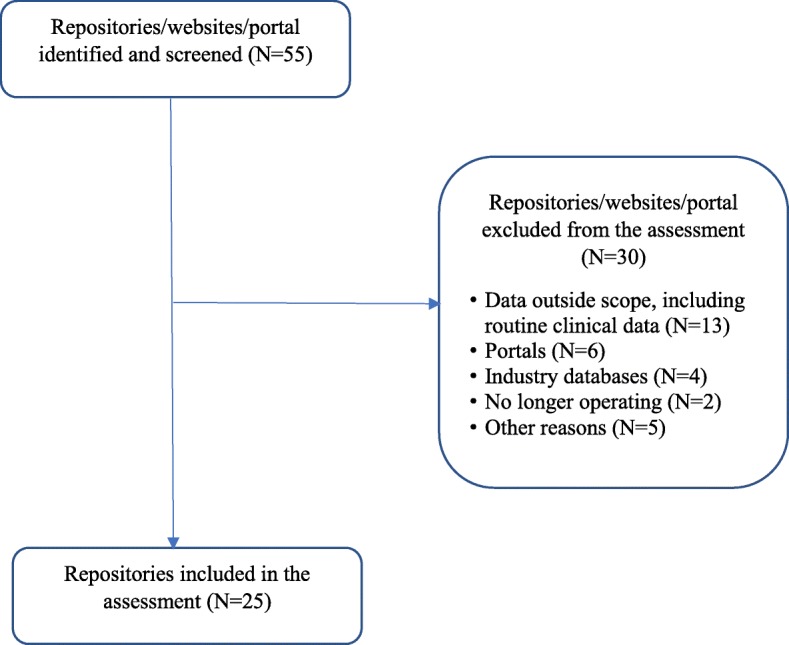
Flow chart

Our search yielded 55 publicly accessible websites of data repositories that appeared to be able to – potentially – host IPD from clinical studies. An initial screening checked each repository for eligibility against the scope of the analysis, which was to describe and assess repositories that enable storage, sharing, discoverability and re-use of IPD from clinical studies and related study documents, for non-commercial researchers. After this screening, 25 repositories which met the eligible criteria were included in the analysis (Fig. 1). Inclusion and exclusion of websites referring to repositories was not always straightforward and required several rounds of discussion. Among those excluded (Additional file 3), were several repositories hosting only data from non-medical fields of research, such as social sciences, legal and forensic disciplines, or ecology (e.g. The Knowledge Network for Biocomplexity, National Archive of Criminal Justice Data). Although, in principle, these repositories might include some sort of health-related research data, it would not make sense to use them as preferred storage option for clinical researchers and thus, we considered them to be outside the scope of our analysis. We also excluded portals and platforms where independent third parties facilitated the interaction between data holders, mainly drug companies, and data users, but which did not store data themselves. We acknowledge that this approach represents an increasingly popular model in data sharing for industry-funded clinical studies and may be applicable also to non-commercial academic trials. For instance, the UK Medical Research Council (MRC) joined Clinicalstudydatarequest.com in April 2018 as part of the academic funder consortium and it is expected that this platform will enable data sharing from MRC-funded clinical trials. We also excluded repositories hosting health data collected in routine clinical practice (such as BioGrid Australia Limited) or population and survey data (such as the UK Data Service).

Table 1 reports the main characteristics of the included repositories (and includes the URLs of their home pages). Half are generic repositories, meaning that they are not limited to a disease or clinical area, and in many cases are not even specific to clinical research, accepting data from a wide range of scientific disciplines. The number of IPD sets stored in the covered repositories varied from none to hundreds. A few repositories, such as those from Early Breast Cancer Trialists’ Collaborative Group, the Immune Tolerance Network, and Vivli, reported the number of clinical studies on their website, but often estimating this number accurately was impossible due to a lack of clear search criteria or appropriate metadata. Four repositories started their activity before 2000, eight between 2000 and 2010, and the remainder were launched after 2010. Most repositories do not apply a fee for uploading, storage or access to data. Notable exceptions are Dryad, Figshare, and Vivli that propose periodic or per-study fees for uploading and publication of data.

**Table 1:** Main characteristics of the included repositories

Name, URL		Scientific scope	Source scope	Host organisation	Main funding	Who can upload data?
Generic						
1	B2Share, https://b2share.eudat.eu/	General scientific	Continental (Europe)	EUDAT centres in various EU countries (Germany, Finland)	Public/Academia (EU Commission and other partners)	Anyone
2	Drum, https://conservancy.umn.edu/handle/11299/166578	General scientific	Institutional	University of Minnesota	Public/Academia (University of Minnesota)	Researcher from University of Minnesota
3	Dryad, https://datadryad.org	General scientific	Global	University of North Carolina, British Library and Oxford University	Not-for-profit membership organisation	Any author of published article
4	EASY, https://easy.dans.knaw.nl/ui/home	General scientific	National (the Netherlands)	Data Archiving and Networked Services	Public/Academia (Royal Netherlands Academy of Arts, Sciences/Organisation for Scientific Research)	Anyone
5	Edinburgh DataShare, https://datashare.is.ed.ac.uk/	General scientific	Institutional	University of Edinburgh	Public/Academia (University of Edinburgh)	Researcher from University of Edinburgh
6	Figshare, https://figshare.com/	General scientific	Global	Figshare (via Amazon web services)	Commercial/ Public/Academia	Anyone from user organisations
7	ICPSR, https://www.icpsr.umich.edu/icpsrweb/ICPSR/index.jsp	Social and health sciences	Global	Institute for Social Research (University of Michigan)	Public/Academia (university consortium)	Anyone
8	Open Science Framework, https://osf.io/	General scientific	Global	Center for Open Science, Virginia (USA)	Private Foundation (Laura and John Arnold Foundation)	Anyone
9	Swedish National Data Service, https://snd.gu.se/en	Social and health sciences	National (Sweden)	University of Gothenburg	Public/Academia (university consortium)	Anyone (mainly Swedish researchers)
10	UMIN, http://www.umin.ac.jp/icdr/	Clinical study	National (Japan)	University Hospital Medical Information Network – University of Tokyo Hospital	Public/Academia (trial registry)	Investigators of trials registered
11	Vivli, https://vivli.org/	Clinical study	Global	Center for global Clinical Research Data, Harvard	Non-profit organisation	Anyone
12	Zenodo, https://zenodo.org	General scientific	Global	CERN (Geneva)	Public/Academia (EU Commission and other partners)	Anyone
Disease specific						
13	CancerData.Org, https://www.cancerdata.org/	Cancer	National (The Netherlands)	Maastro Clinic (The Netherlands)	Health care corporate	*Not clear*
14	Project Datasphere, https://www.projectdatasphere.org/projectdatasphere/html/home	Cancer	Global	CEO Roundtable on Cancer, Inc. (USA)	Pharma consortium	Researchers/pharma in the field of cancer
15	EBCTCG, https://www.ctsu.ox.ac.uk/research/ebctcg	Breast cancer	Global	Oxford University (UK)	Public/Academia (UK Medical Research Council and Cancer Research)	Early Breast Cancer Trialists’ Collaborative Group
16	FreeBird, https://ctu-app.lshtm.ac.uk/freebird/	Injury and emergency medicine	Global	London School of Hygiene & Tropical Medicine (UK)	Public/Academia	Researchers in the field of emergency medicine
17	ITN Trialshare, https://www.immunetolerance.org/researchers/trialshare	Allergy, Asthma, Autoimmune disease, Transplantation,	National (USA) – collaborative network	National Institute of Allergy and Infectious Diseases, NIH (USA)	Public/Academia (NIH)	Researchers of ITN promoted trial
18	Melanoma MMP, http://www.mmmp.org/MMMP/public/trials/listTrials.mmmp	Melanoma	Global	*Not clear*	Public/Academia (non-profit association)	Apparently anyone (melanoma)
19	NDACAN, www.ndacan.cornell.edu/	Child abuse and neglected	National (USA)	National Data Archive on Child Abuse and Neglect (Cornell University)	Public/Academia (Children’s Bureau and Department of Health and Human Services)	Anyone (child abuse research)
20	NDCT NIMH, https://data-archive.nimh.nih.gov/ndct	Mental health	National (USA)	National Institute of Mental Health	Public/Academia (NIH)	Anyone (mental health-related clinical trial)
21	NIDDK, https://repository.niddk.nih.gov/home/	Diabetes, digestive, and kidney diseases	National (USA)	National Institute of Diabetes and Digestive and Kidney Diseases	Public/Academia (NIH)	Researchers of NIDDK-funded studies
22	NIH BioLINCC, https://biolincc.nhlbi.nih.gov/home/	Heart, lung, blood diseases	National (USA)	National Heart, Lung, and Blood Institute	Public/Academia (NIH)	Researchers of NHLBI funded-project
23	ProAct, https://nctu.partners.org/ProACT	Amyotrophic lateral sclerosis	Global	Prize4Life Israel & Massachusetts General Hospital	Not-for-profit/Pharma	Data donated by project partners
24	TBI-IMPACT, http://www.tbi-impact.org/	Traumatic brain injury	Global	Antwerp, Rotterdam and Edinburgh	Public/Academia	*Not clear*
25	WWARN, www.wwarn.org/	Malaria and other infectious diseases	Global	University of Oxford	Public/Charity	Anyone (malaria)

Acronyms: *CERN* Conseil européen pour la recherche nucléaire; *DRUM* Data Repository for the University of Minnesota; *EASY* Electronic Archiving System; *EBCTCG* Early Breast Cancer Trialists’ Collaborative Group; *ITN* Immune Tolerance Network; *NDACAN* National Data Archive on Child Abuse and Neglect; *NDCT* National Database for Clinical Trials Related to Mental Illness; *NHLBI* National Heart, Lung, and Blood Institute; *NIDDK* National Institute of Diabetes and Digestive and Kidney Diseases; *NIH* National Institute of Health; *NIMH* National Institute of Mental Health; *TBI* Traumatic brain injury, *UMIN* University Medical Information Network; *WWAAR* WorldWide Antimalarial Resistance Network

Although the sample is highly heterogeneous, it is possible to delineate a group of repositories developed under the auspices of public research funders and infrastructures. For instance, in Europe, B2SHARE, funded by EUDAT, and Zenodo, funded by the European Organization for Nuclear Research (Conseil européen pour la recherche nucléaire: CERN) are part of a data-sharing infrastructure providing data services for researchers, scientific communities, institutions, and even citizen scientists to store and exchange data from various disciplines. In the United States, an interesting subgroup of repositories is formed by several NIH-funded institutes. Since 2003, the NIH has required researchers to share the underlying findings from the data from any research project with NIH grants exceeding US$500,000 [9]. This requirement prompted the development of disease-specific data repositories that primarily, but not exclusively, host data from NIH-funded projects in a given disease area, e.g. mental health, autism, drug abuse, to support the release and sharing of final research data from NIH-supported studies for use by other researchers. The Trans-NIH BioMedical Informatics Coordinating Committee lists about 70 data repositories, most accepting submissions of appropriate data from NIH-funded investigators (and others), though some are used only for a specific research network or programme. Only some of them host IPD data from clinical studies, and, although they are not completely homogenous, they share some common features as they all refer to overall NIH policies for data preparation and dissemination, including anonymisation [26]. Usually these repositories promote models of access that are open by default with restrictions or embargos possible for some type of contents. Data are usually stored and accessed for free as costs are covered by funders in the project main funding.

Other repositories focus on services for sharing data that support the findings reported in journal articles and other scholarly publications. Such repositories may gain in importance, as medical journals are increasingly demanding a commitment to share data collected in clinical trials [15]. For instance, in Dryad, all material is associated with a scholarly publication. The repository covers all research areas, including medicine, and hosts IPD from clinical studies published in medical journals. Similarly, Figshare is a repository system, available both as a Cloud-hosted service or integrated with local storage, that provides services for storing and make visible data objects linked to the published data of several scientific publishers, such as PLoS, F1000, Nature, and Wiley. Figshare is also one of the systems providing data repositories for individual universities, often as a hosted service and sometimes integrated with local university systems. Universities increasingly rely on data repositories to improve the visibility of their research output with institution specific repositories, usually restricting upload of data and documents to the university’s own staff. The Data Repository for the University of Minnesota (DRUM) and Edinburgh DataShare are two examples included in our analysis, although several other academic repositories may be available to store IPD [27].

Three repositories were identified with a national scope, in terms of the source of the data and those able to upload it: UMIN (University Medical Information Network) in Japan, EASY (Electronic Archiving System) in the Netherlands, and the Swedish National Data Service (SND), though the latter has accepted a small proportion of data from elsewhere. In addition, the NIH repositories described earlier are clearly mostly, if not exclusively, intended for US-based researchers.

**Fig. 2:**
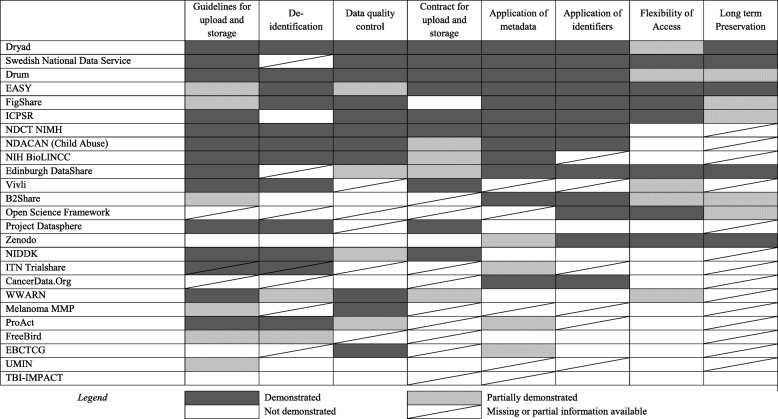
Suitability of the repository for hosting clinical study data

Figure 2 is an attempt to summarise the suitability of each repository for hosting IPD from clinical studies, by evaluating it against the key attributes listed earlier (see Additional file 2 for details). None of the repositories demonstrated completely all the items we included in the indicator set; however, three repositories (Dryad, DRUM, EASY) were judged as demonstrating or partially demonstrating all the items. All three are generic repositories. In the case of four repositories (SND, Edinburgh DataShare, Vivli, and Open Science Framework) we did not judge any of the considered items as not demonstrated, but we were not able to retrieve the information needed to judge one or more items. The remaining repositories appeared to be less suitable for hosting clinical study data, although our analysis is limited by the fact that, in several cases, we could not retrieve enough information to allow an accurate judgment. For example, information on arrangements for the long-term preservation was not publicly reported in more than half of the analysed repositories. This information, which for many depositors might be an important factor in driving the choice of one repository over another, could usefully be displayed explicitly on the website.

## Discussion

### Summary of main findings

The study has shown that an increasing number of repositories are available for sharing of IPD from clinical studies. There are many different types of repositories, such as generic repositories for all kinds of life-science data, generic repositories exclusively for clinical research data and specific repositories for study data with a specific focus (e.g. disease-specific, stakeholder-specific, institutional-specific). Only two repositories may be considered ‘general clinical research’, the Japanese National Repository linked to the National Registry for Clinical Trials (UMIN), and Vivli that was launched in 2018. This scenario may reflect the fact that data sharing in clinical research – whilst encouraged and accepted in principle by many for some time [1–4, 22, 28] – has only been given a strong push towards implementation in recent years, especially with data-sharing requirements defined by funders [9–11] and journal editors [15] changing the expectations about data release. There may, therefore, be a much greater demand for repositories of this sort in the future – perhaps at a national or continental level, as well as for better support for clinical research data objects in general and institutional systems.

Major heterogeneity exists with respect to data-upload, data-handling, and data-access processes. Our attempt to apply a measure to estimate the repository suitability for clinical study data was only approximate because of the paucity of information publicly available, but even so it did reveal major differences between repositories. Only a few repositories appeared to exhibit all or most of the suitability indicators (Fig. 2). The heterogeneity, of both repository types and features, reflects the different purposes and perspectives of repository founders, and the relative immaturity of repository data-sharing services. Given the lack of a consensus about the services required from a data repository, each organisation has implemented its own policies and systems to meet its own priorities. This may create major hurdles if datasets from different repositories are needed for a research project. The disease-specific repositories have often evolved from particular collaborations or research communities; the more general repositories from conscious decisions by funders, academic organisations and occasionally governments aiming at supporting ‘open science’. The data suggest that the availability of different options for data access could be improved. Only seven of the 25 repositories offered two or more clear choices, though a further five did offer a limited choice (e.g. adding an embargo period to open access, or two different forms of managed access). Clinical study datasets may carry different residual risks for re-identification, or be backed by different forms of consent about data sharing. We therefore believe that having options available to data depositors to select, for instance, open access with self-attestation of the user, or managed access following review, or a granular access to different parts of the datasets, is important.

### Strengths and limitations of the study

We provided a detailed description of repositories and assessed their suitability for clinical research data objects, from the perspective of clinical researchers using criteria selected specifically for that purpose. Two independent assessors evaluated the publicly available information about each repository, as to reduce the risks of possible errors in the data extraction, and misinterpretation. It is an initial attempt to collect and disseminate practical information for researchers who are increasingly faced with the question of where they should deposit their data in the long term, and proved useful in clarifying the numbers and types of repositories available and the facilities they offered.

Our evaluation is a snapshot of what is a rapidly evolving landscape. Despite our attempts to identify all the possible candidates to be included in our analysis we could have missed important initiatives, such as those promoted in languages other than English or those not having a strong web presence. Thus, we do not claim that the sample of repositories is complete, though we believe that it is representative of the current situation. Though we started by evaluating a relatively large sample of candidate repositories (55), the scoping rule (suitability for data deposition by non-commercial researchers) resulted in a final sample of 25. Platforms that enable access to industry trial datasets, such as CSDR and YODA, now also list studies from non-commercial sponsors, so they may become valuable options too.

We based our evaluation of repository features on an arbitrary indicator set intended to indicate the likelihood that data and documents would be stored securely but flexibly and shared appropriately. This analysis should be interpreted as an initial attempt to assess the maturity of repositories for this purpose and not a final judgement. We would welcome a dialogue with other interested parties, including repositories, in developing these assessment criteria and methods further. The elaboration of this indicator set was informed by what the team of authors believe is the most relevant to clinical researchers seeking a repository for their data. We collected inputs from a wider discussion in the context of a consensus exercise on IPD sharing and re-use [5] and from examining other existing systems for assessing the quality of data repositories [23]. We felt that none of the existing assessment schemes exactly fitted our needs. For instance, the CoreTrustSeal criteria, though informative, appeared to be too general for our purposes [23]. High-level criteria specifically designed for clinical research data have been proposed [29, 30] but, in both cases, they are useful lists of desirable features but difficult to assess against without further details of the criteria being available. This study and its results may inform programmes that attempt to integrate available certification systems, with extensions where necessary to repositories in specific domains. This may help to assess and certify the core components of a FAIR data ecosystem in clinical research [31].

Not all of the data and documents needed to evaluate the repositories were always available openly on the website or after simple registration. This was especially the case for information about funding and sustainability of repositories. As a consequence, we may have misclassified some of the features we assessed, because of the lack of proper information. We hope our analysis may serve to improve the self-description of data repositories, to allow a user to access more complete information and help to inform the choice of a particular data repository. In the context of open science having access to sufficient metadata, descriptions, and documentation is as important as having access to the datasets themselves [31] and repositories should be encouraged to present their usage rules and sustainability information as openly as possible to the researcher.

### Findings in the context and implications for future research

Currently, there is a clear tendency for clinical study data to be made more openly available, to allow its re-use, and several stakeholders and constituencies are promoting this change. They share the view that responsible data sharing will promote better research, increasing visibility and reducing duplication [1–5, 32]. It can be expected that data repositories will play an essential role in increasing the accessibility and reusability of research data. The complex evolution of repositories’ features and its impact on research and health deserves periodic or ongoing assessment applying, for instance, methodologies typical of observatories or natural experiment [28].

A few studies have reported on the researchers’ experience and concerns of sharing data through repositories. About 70% of the clinical trial units responding to a survey done in 2015 in the UK would be prepared, in principle, to transfer data to a central repository [33]. Rathi et al. surveyed authors of clinical trials published in 2010 or 2011 in the six highest-impact general medical journals and found that only 18% of the respondents were required to deposit their trial data in a repository, and of these, half had actually done so [34]. Respondents were also asked about concerns with sharing data through repositories, and more than three quarters reported actual or hypothetical concerns, mainly concerning the misuse of data [35]. Other analyses explored the actual commitment to IPD sharing. Very few (5%) of the studies registered at ClinicalTrials.gov in 2016–2017 by high-volume registrants provided a data-sharing plan [36] and only 4% of the consent forms of a sample of antibiotic studies, both from public and industry funders, reported a commitment to share IPD [37].

More consistent and clear procedures and processes between repositories could help to mitigate these concerns, bearing in mind that the number of repositories is increasing. Currently support for features (such as the details of different access options) that would be of particular value to clinical researchers seeking a repository is relatively limited, but it might be possible to improve this using the agreed suitability criteria as additional data points. An important extension of that collaboration would relate to sharing metadata about repository contents, using a common metadata schema and pooling those metadata into a ‘metadata repository’ [38].

The effort to standardise data-sharing practices would have to confront issues that are wider than the research environment, including for instance, questions surrounding legal compliance to national or regional privacy regulations for the storage and re-use of data simply defined as ‘de-identified’. In dealing with these issues, data repositories may play a role in improving the support for managing sensitive datasets and, specifically, clinical research data.

The process of defining a clear description of a ‘high-quality repository’ for clinical research data is still in its initial stages; a set of suitability standards or indicators built on the standards already proposed would need to be specific enough to be assessable and be agreed by the key stakeholders (e.g. research producing organisations, funders, and repositories) as appropriate. Standard instruments (e.g. checklists) for assessment of maturity and suitability of repositories could be of major help. Resource constraints would mean that any assessment process would probably involve the repositories themselves constructing an evidence portfolio against the criteria, similar to the current CoreTrustSeal scheme for general repositories [23]. In our sample of repositories, only a few (e.g. DRUM, Edinburgh DataShare, EASY) appeared to have this certification, suggesting possible difficulties in its implementation.

### Final remarks

Our study provides a detailed – though preliminary – picture of the available repositories that may, in principle, host clinical study data. Some useful conclusions and implications for future works can be drawn; however, it seems too early to provide a conclusive answer to the central question of the study – where could non-commercial researchers store their datasets? With few exceptions, e.g. a researcher working in an area served by a disease-specific repository or in a country that has a national repository service available, the choice is likely to involve a trade-off between different desirable features. The use of data repositories can bring several advantages to the data depositor, such as the provision of secure long-term management of the data long after the original research team has disbanded, the application of identifiers to all material so that it is accurately cited and the original data generators are properly acknowledged when data are re-used, help in preparing data for sharing, and in managing the sometimes complex processes of applying for and granting access. The final goal should be an improvement and harmonisation of practices of repositories that may diminish the reluctance of many researchers to share the data of their studies, thus promoting data-sharing, discoverability, and re-use.

## Additional files

Additional file 1: List of items for data collection. (DOCX 29 kb)

Additional file 2: Details on indicator elaboration. (ZIP 35 kb)

Additional file 3: List of excluded websites. (ZIP 35 kb)

Additional file 4: Data underlying the results reported in the manuscript. (XLSX 88 kb)
