# Supplementary material for: Evaluation of repositories for sharing individual-participant data from clinical studies
Source: Trials. 2019 Mar 15;20:169. doi: 10.1186/s13063-019-3253-3 (PMC6420770; doi:10.1186/s13063-019-3253-3)
Supplement: Supplementary file 1 — List of items for data collection. (DOCX 29 kb) [file 13063_2019_3253_MOESM1_ESM.docx]

Additional file 1: **Table S1** list of items for data collection

| **Code** | **Name** | **Information sought** |
| --- | --- | --- |
| 1. **General Parameters** | | |
| A1 | Name | Name of the repository |
| A2 | URL (Website) | URL of the repository’s home page |
| A3 | Location | Physical location(s) of the repository, including host organisation(s) |
| A4 | Self-description, (max. ½ page) | As from the web site, main features /claims |
| A5 | Scientific scope | 1 = General scientific, 2 = Social and health sciences, 3 = Clinical&epidemiology studies, 4 = Clinical trials only, 5 = Specified study or group of studies, 6 = Other |
| A6 | Source scope (geo-coverage) | In terms of the location of the source material: 1 = Global, no restriction, 2 = Continental, 3 = National, 4 = Regional (within a country), 5 = Institution or Organisation, 6 = Other |
| A7 | R3data | Yes if listed in R3data registry |
| A8 | Start year | The year the repository started (in its current form or similar) |
| A9 | Main funding | 1 = public / academia, 2 = pharma industry,  3 = academia / public and pharma consortium,  4 = Other |
| 1. A10 | Repository sustainability | Information on the funding position of the repository in the longer term |
| 1. A11 | Business continuity | Information on the commitment to preservation of the data if the repository has to close? (Y/N, unknown) |
| 1. **Data Upload and Storage** | | |
| 1. B1 | Rules and guidelines on upload | Does the repository have and apply rules and/or guidelines for uploading data? (Y/N, unknown) |
| 1. B2 | Who can upload data | Restrictions on who can upload, if any |
| 1. B3 | Format, metadata and documentation requirements for upload? | Are there particular ways in which data must be formatted, and / or metadata and documentation provided? (Y/N, unknown) |
| 1. B4 | De-identification practices before upload | Are there particular requirements or guidelines relating to the de-identification of uploaded data? (Y/N, unknown) |
| 1. B5 | Control of quality of data | Are there control/review mechanisms in place to check data quality when it is submitted to the repository? (Y/N, unknown) |
| 1. B6 | Acceptable size of files | Is there a limit to the size of files and datasets that can be uploaded (Y/N, unknown) |
| B7 | Costs of upload | Is there any cost associated with uploading data charged to the data generator or depositor (Y/N, unknown) |
| B8 | Formal contract regarding upload and storage | Is there a formal agreement to be signed by the data generator and repository specifying the roles and responsibilities of each? (Y/N, unknown) |
| B9 | Costs of storage | Is there any cost associated with maintaining data in the repository, to the data generator (Y/N, unknown) |
| 1. B10 | Length of storage | Are there any limits on the storage period of uploaded data, or is it viewed as indefinite? |
| 1. **Data Available and Access** | | |
| C1 | Has public clinical study data (without self-attestation)? | Whether has clinical study data that can be accessed and downloaded by the public, *without any user self-attestation;* 1 = Yes, 2 = No, and would not, 3 = No but might in the future, 4 = Unknown, 5= No |
| C2 | Has public clinical study data (following self-attestation) | Whether has clinical study data that can be accessed and downloaded by the public, *following web-based user self-attestation;* 1 = Yes, 2 = No, and would not, 3 = No but might in the future, 4 = Unknown, 5= No |
| C3 | Has managed access clinical study data? | Whether has clinical study data with controlled access or a range of different access types, to a files, e.g. through group membership or case-by-case review; embargo as example of minimal type of managed access.  1 = Yes, 2 = No, and would not, 3 = No but might in the future, 4 = Unknown, 5 = No |
| C4 | Managed access to analysis environment | Whether has clinical study data that can only be accessed in situ for analysis purposes  1 = Yes, 2 = No, and would not, 3 = No but might in the future, 4 = Unknown, 5 = No |
| C5 | Total number of clinical studies involved | If the repository has clinical study data (B1, B2, B3 or B4), approximately how many studies have generated that data? |
| C6 | Other clinical study data objects | Whether the repository includes other clinical study documents and data, e.g. protocols, CSRs, analysis plans etc.; 1 = Yes, 2 = No, and would not, 3 = No but might in the future, 4 = Unknown, 5 = No |
| 1. **Discoverability** | | |
| 1. D1 | Application of an identifier | Are identifiers (e.g. a DOI) assigned to datasets and other material stored within the repository?  1 = Yes (assigned before or after upload), 2 = Mixed, some material has identifiers, some not, 3 = No, 4 = Unknown |
| 1. D2 | Application of a metadata schema to describe contents | Does the repository use a consistent metadata schema (or schemas) to describe its contents, either internally, on web pages, or both? (Y/N) |
| 1. D3 | Metadata availability on the web | Does the repository provide a web-based catalogue of its contents, searchable by humans (a so-called metadata repository)? (Y/N, unknown) |
| 1. D4 | Metadata availability through an API | Does the repository provide an API for machine-based interrogation of its metadata / catalogue? (Y/N, unknown) |
| D5 | Access details available for secondary users | Are guidelines/policies about (controlled) secondary access available to users, for example how to apply and to whom? (Y/N, unknown) |
| D6 | Costs to user? | Does gaining access to data or other data objects involve a cost to the secondary user, at least in some cases? (Y/N, unknown) |
| 1. D7 | Format of accessed data | Is data downloaded or accessed in situ only available in particular formats?  1 = No, data downloaded / accessed is unchanged from original uploaded format (any format may be found), 2 = Yes, data available in particular formats only, 3 = Other |
