# Supplementary material for: Evaluation of repositories for sharing individual-participant data from clinical studies
Source: Trials. 2019 Mar 15;20:169. doi: 10.1186/s13063-019-3253-3 (PMC6420770; doi:10.1186/s13063-019-3253-3)
Supplement: Supplementary file 2 — Details on indicator elaboration. (ZIP 35 kb) [file 13063_2019_3253_MOESM2_ESM.zip › Additional file 2_revR1.docx]

**Additional file 2: Details on the elaboration of the maturity for clinical study data indicator (demonstrated, partly demonstrated, not demonstrated, missing or incomplete data)**

1. **Guidelines for data Upload and Storage**

Demonstrated: if the repositories have and apply rules and/or guidelines for uploading data, i.e. genuine guidelines on preparing the data, formats, metadata etc.

Partially demonstrated: if only simple ‘how-to upload’ instructions’ is available.

Not demonstrated: if no rules and/or guidelines for uploading data are available.

Missing or incomplete data: not enough details available in the public website to judged

1. **De-identification practices before upload**

Demonstrated: if the repository has requirements or guidelines relating to the de-identification of uploaded data and explicit information or links are provided.

Partially demonstrated: if generic terminology about the requirement to upload de-identified data is used but no explicit information is available.

Not demonstrated: if no de-identification process or check is implemented.

Missing or incomplete data: not enough details available in the public website to judged.

1. **Control of quality of data**

Demonstrated: if the repositories have and apply control and review mechanisms to check data when submitted, including for instance formatting, completeness, de-identification.

Partially demonstrated: if only a simple formatting curation is implemented.

Not demonstrated: if data can be submitted “as it is”.

Missing or incomplete data: not enough details available in the public website to judged.

1. **Formal contract regarding upload and storage**

Demonstrated: if a formal agreement specifying the roles and responsibilities needs to be signed by the data generator and repository before uploading.

Partially demonstrated: if only a generic “terms of use” for using the repositories needs to be signed by the data generator and repository before uploading.

Not demonstrated: if no agreement/contract is implemented.

Missing or incomplete data: not enough details available in the public website to judged.

1. **Application of a metadata schema to describe contents**

Demonstrated: if the repository uses a consistent and detailed metadata schema (or schemas) to describe its contents, internally, on web pages, or on both.

Partially demonstrated: if metadata requirements are limited (for instance, only requiring name of the study, description, authors.

Not demonstrated: if no metadata scheme is applied.

Missing or incomplete data: not enough details available in the public website to judged

1. **Application of an identifier**

Demonstrated: if identifiers (e.g. a DOI) is assigned to datasets and other material stored within the repository.

Partially demonstrated: not applicable

Not demonstrated: if no identifiers is applied.

Missing or incomplete data: not enough details available in the public website to judged

1. **Flexibility of access**

Demonstrated: if the repository allows the choice between two or more different options for making data accessible, for instance open with self-attestation of the user, or managed access, granular access to different part of the datasets, etc. The decision of one option over the others may be driven by the residual risks for re-identification, contents of the informed consent, types of requests, etc.

Partially demonstrated: if the repository allows a limited range of access types as open access with embargo periods, or different modalities of restricted access (e.g., data access committee or direct managed control).

Not demonstrated: if only one access modality is available, for instance managed access or open access with attestation only.

Missing or incomplete data: not enough details available in the public website to judged.

1. **Repository long term preservation**

Demonstrated: if the repository has, explicitly, guaranteed information for a set period, plus explicit continuity measures.

Partially demonstrated: if the repository has no guaranteed funding but does have business continuity measures in place.

Not demonstrated: if funding appears to have ceased.

Missing or incomplete data: not enough details available in the public website to judged.
