# Supplementary material for: Evaluation of repositories for sharing individual-participant data from clinical studies
Source: Trials. 2019 Mar 15;20:169. doi: 10.1186/s13063-019-3253-3 (PMC6420770; doi:10.1186/s13063-019-3253-3)
Supplement: Supplementary file 3 — List of excluded websites. (ZIP 35 kb) [file 13063_2019_3253_MOESM3_ESM.zip › Additional file 3_revR1.docx]

Additional file 3: Table S3: List of excluded repositories/portals/websites with reasons

|  | **Name** | **Main reason for exclusion** |
| --- | --- | --- |
| 1 | BioGrid Australia Limited | Routine clinical data |
| 2 | CTTI: Clinical Trials Transformation Initiative | Portal, not a repository |
| 3 | UFIDR: University of Florida Health Integrated Data Repository | Routine clinical data |
| 4 | YODA: The Yale University Open Data Access Project | Platform |
| 5 | Clinicalstudydatarequest.com | Platform, not a repository |
| 6 | Fairsharing (formerly biosharing) | Platform with standards and policies |
| 7 | BIRN: Biomedical Informatics Research Network | No longer operating |
| 8 | SOAR: Duke Clinical research Institute | Industry trials + routine clinical data |
| 9 | DataOne | Data outside scope (earth science data) |
| 10 | Critical Path Institute | Portal, not a repository |
| 11 | IST: International Stroke Trial Database (Edinburgh) | Part of Edinburgh DataShare |
| 12 | NDAR: National Database for Autism Research (US) | Part of NIMH NDCT |
| 13 | NIH NIDA: National Institute of Drug Abuse (US) | Institutional website, not a repository |
| 14 | Substance Abuse and Mental Health Data Archive | Data outside scope (survey and population data) |
| 15 | Sylvia Lawry Centre | Institutional website, no longer operating |
| 16 | Pfizer clinical trial data | Industry trial only |
| 17 | Roche trials | Industry trial only |
| 18 | Global Health Data Exchange | Population data |
| 19 | Henry A. Murray Research Archive at Harvard University | Social sciences data only |
| 20 | INDEPTH Data Repository | Population data |
| 21 | National Archive of Criminal Justice Data | Data outside scope (legal and forensic data) |
| 22 | Neuroscience Information Framework | Portal, not a repository |
| 23 | The Knowledge Network for Biocomplexity | Data outside scope (ecology data) |
| 24 | UK Data Service | Data outside scope (survey and population data) |
| 25 | UK Data Archive | Data outside scope (UK social science data) |
| 26 | Health and Medical Care Archive | Data from Robert Wood Johnson Foundation projects |
| 27 | National Addiction & HIV Data Archive Program | Data outside scope |
| 28 | National Archive of Computerized Data on Aging | Data outside scope |
| 29 | FDA Janus | SDTM based FDA submission data only |
| 30 | Run My Code | Project management tool |
